# Inherited variants affecting RNA editing may contribute to ovarian cancer susceptibility: results from a large-scale collaboration

**DOI:** 10.18632/oncotarget.10546

**Published:** 2016-07-12

**Authors:** Jennifer B. Permuth, Brett Reid, Madalene Earp, Y. Ann Chen, Alvaro N.A. Monteiro, Zhihua Chen, AOCS Study Group, Georgia Chenevix-Trench, Peter A. Fasching, Matthias W. Beckmann, Diether Lambrechts, Adriaan Vanderstichele, Els Van Niewenhuyse, Ignace Vergote, Mary Anne Rossing, Jennifer Anne Doherty, Jenny Chang-Claude, Kirsten Moysich, Kunle Odunsi, Marc T. Goodman, Yurii B. Shvetsov, Lynne R. Wilkens, Pamela J. Thompson, Thilo Dörk, Natalia Bogdanova, Ralf Butzow, Heli Nevanlinna, Liisa Pelttari, Arto Leminen, Francesmary Modugno, Robert P. Edwards, Roberta B. Ness, Joseph Kelley, Florian Heitz, Beth Karlan, Jenny Lester, Susanne K. Kjaer, Allan Jensen, Graham Giles, Michelle Hildebrandt, Dong Liang, Karen H. Lu, Xifeng Wu, Douglas A. Levine, Maria Bisogna, Andrew Berchuck, Daniel W. Cramer, Kathryn L. Terry, Shelley S. Tworoger, Elizabeth M. Poole, Elisa V. Bandera, Brooke Fridley, Julie Cunningham, Stacey J. Winham, Sara H. Olson, Irene Orlow, Line Bjorge, Lambertus A. Kiemeney, Leon Massuger, Tanja Pejovic, Melissa Moffitt, Nhu Le, Linda S. Cook, Angela Brooks-Wilson, Linda E. Kelemen, Jacek Gronwald, Jan Lubinski, Nicolas Wentzensen, Louise A. Brinton, Jolanta Lissowska, Hanna Yang, Estrid Hogdall, Claus Hogdall, Lene Lundvall, Paul D.P. Pharoah, Honglin Song, Ian Campbell, Diana Eccles, Iain McNeish, Alice Whittemore, Valerie McGuire, Weiva Sieh, Joseph Rothstein, Catherine M. Phelan, Harvey Risch, Steven Narod, John McLaughlin, Hoda Anton-Culver, Argyrios Ziogas, Usha Menon, Simon Gayther, Susan J. Ramus, Aleksandra Gentry-Maharaj, Celeste Leigh Pearce, Anna H. Wu, Jolanta Kupryjanczyk, Agnieszka Dansonka-Mieszkowska, Joellen M. Schildkraut, Jin Q. Cheng, Ellen L. Goode, Thomas A. Sellers

**Affiliations:** ^1^ Department of Cancer Epidemiology, Moffitt Cancer Center, Tampa, FL, USA; ^2^ Department of Health Science Research, Division of Epidemiology, Mayo Clinic, Rochester, MN, USA; ^3^ Department of Biostatistics and Bioinformatics, Moffitt Cancer Center, Tampa, FL, USA; ^4^ Genetics and Computational Biology Department, QIMR Berghofer Medical Research Institute, Queensland, Australia; ^5^ Cancer Genetics Laboratory, Research Division, Peter MacCallum Cancer Centre, Melbourne, Australia; ^6^ David Geffen School of Medicine, Department of Medicine, Division of Hematology and Oncology, University of California at Los Angeles, Los Angeles, CA, USA; ^7^ University Hospital Erlangen, Department of Gynecology and Obstetrics, Friedrich-Alexander-University Erlangen-Nuremberg, Comprehensive Cancer Center, Erlangen, Germany; ^8^ Vesalius Research Center, VIB, Leuven, Belgium; ^9^ Laboratory for Translational Genetics, Department of Oncology, University of Leuven, Leuven, Belgium; ^10^ Division of Gynecologic Oncology, Department of Obstetrics and Gynaecology and Leuven Cancer Institute, University Hospitals Leuven, Leuven, Belgium; ^11^ Program in Epidemiology, Division of Public Health Sciences, Fred Hutchinson Cancer Research Center, Seattle, WA, USA; ^12^ Department of Epidemiology, University of Washington, Seattle, WA, USA; ^13^ Department of Epidemiology, Geisel School of Medicine, Dartmouth College, Hanover, NY, USA; ^14^ German Cancer Research Center, Division of Cancer Epidemiology, Heidelberg, Germany; ^15^ University Cancer Center Hamburg (UCCH), University Medical Center Hamburg-Eppendorf, Hamburg, Germany; ^16^ Department of Cancer Prevention and Control, Roswell Park Cancer Institute, Buffalo, NY, USA; ^17^ Department of Gynecologic Oncology, Roswell Park Cancer Institute, Buffalo, NY, USA; ^18^ Cancer Prevention and Control, Samuel Oshin Comprehensive Cancer Institute, Cedars-Sinai Medical Center, Los Angeles, CA, USA; ^19^ Community and Population Health Research Institute, Department of Biomedical Sciences, Cedars-Sinai Medical Center, Los Angeles, CA, USA; ^20^ Cancer Epidemiology Program, University of Hawaii Cancer Center, Honolulu, HI, USA; ^21^ Gynaecology Research Unit, Hannover Medical School, Hannover, Germany; ^22^ Radiaton Oncology Research Unit, Hannover Medical School, Hannover, Germany; ^23^ Department of Pathology, University of Helsinki and Helsinki University Hospital, Helsinki, Finland; ^24^ Department of Obstetrics and Gynecology, University of Helsinki and Helsinki University Central Hospital, Helsinki, Finland; ^25^ Division of Gynecologic Oncology, Department of Obstetrics, Gynecology and Reproductive Sciences, University of Pittsburgh School of Medicine, Pittsburgh, PA, USA; ^26^ Ovarian Cancer Center of Excellence, Womens Cancer Research Program, Magee-Womens Research Institute & University of Pittsburgh Cancer Institute, Pittsburgh, PA, USA; ^27^ Department of Epidemiology, University of Pittsburgh Graduate School of Public Health, Pittsburgh, PA, USA.; ^28^ The University of Texas School of Public Health, Houston, TX, USA; ^29^ Department of Gynecology and Gynecologic Oncology, Kliniken Essen-Mitte/Evang. Huyssens-Stiftung/Knappschaft GmbH, Essen, Germany; ^30^ Department of Gynecology and Gynecologic Oncology, Dr. Horst Schmidt Klinik Wiesbaden, Wiesbaden, Germany; ^31^ Women's Cancer Program at the Samuel Oschin Comprehensive Cancer Institute, Cedars-Sinai Medical Center, Los Angeles, California, USA; ^32^ Department of Virus, Lifestyle and Genes, Danish Cancer Society Research Center, Copenhagen, Denmark; ^33^ Department of Gynaecology, Rigshospitalet, University of Copenhagen, Copenhagen, Denmark; ^34^ Cancer Epidemiology Centre, Melbourne, Australia; ^35^ Centre for Epidemiology and Biostatistics, University of Melbourne, Australia; ^36^ Department of Epidemiology and Preventive Medicine, Monash University, Melbourne, Australia; ^37^ Department of Epidemiology, The University of Texas MD Anderson Cancer Center, Houston, TX, USA; ^38^ College of Pharmacy and Health Sciences, Texas Southern University, Houston, TX, USA; ^39^ Department of Gynecologic Oncology, The University of Texas MD Anderson Cancer Center, Houston, TX, USA; ^40^ Gynecology Service, Department of Surgery, Memorial Sloan Kettering Cancer Center, New York, NY, USA; ^41^ Department of Obstetrics and Gynecology, Duke University Medical Center, Durham, NC, USA; ^42^ Obstetrics and Gynecology Epidemiology Center, Brigham and Women's Hospital, Boston, MA, USA; ^43^ Department of Epidemiology, Harvard T.H. Chan School of Public Health, Boston, MA, USA; ^44^ Channing Division of Network Medicine, Brigham and Women's Hospital and Harvard Medical School, Boston, MA, USA; ^45^ Cancer Prevention and Control Program, Rutgers Cancer Institute of New Jersey, New Brunswick, NJ, USA; ^46^ Department of Biostatistics, University of Kansas Medical Center, Kansas City, KS, USA; ^47^ Memorial Sloan Kettering Cancer Center, Department of Epidemiology and Biostatistics, New York, NY, USA; ^48^ Centre for Cancer Biomarkers, Department of Clinical Science, University of Bergen, Bergen, Norway; ^49^ Department of Gynecology and Obstetrics, Haukeland University Hospital, Bergen, Norway; ^50^ Radboud University Medical Center, Radboud Institute for Health Sciences, Nijmegen, Netherlands; ^51^ Radboud University Medical Center, Radboud Institute for Molecular Life Sciences, Department of Gynaecology, Nijmegen, The Netherlands; ^52^ Department of Obstetrics and Gynecology, Oregon Health and Science University, Portland, OR, USA; ^53^ Knight Cancer Institute, Oregon Health and Science University, Portland, Oregon, USA; ^54^ Cancer Control Research, BC Cancer Agency, Vancouver, BC, Canada; ^55^ Division of Epidemiology and Biostatistics, Department of Internal Medicine, University of New Mexico, Albuquerque, NM, USA; ^56^ Canada's Michael Smith Genome Sciences Centre, BC Cancer Agency, Vancouver BC, Canada; ^57^ Department of Biomedical Physiology and Kinesiology, Simon Fraser University, Burnaby, BC, Canada; ^58^ Department of Public Health Sciences, Medical University of South Carolina College of Medicine, Charleston, SC, USA; ^59^ International Hereditary Cancer Center, Department of Genetics and Pathology, Pomeranian Medical University, Szczecin, Poland; ^60^ Division of Cancer Epidemiology and Genetics, National Cancer Institute, Bethesda, MD, USA; ^61^ Department of Cancer Epidemiology and Prevention, The Maria Sklodowska-Curie Memorial Cancer Center, Warsaw, Poland; ^62^ The Juliane Marie Centre, Department of Obstetrics and Gynecology, Rigshospitalet, Copenhagen, Denmark; ^63^ Department of Public Health and Primary Care, University of Cambridge, Strangeways Research Laboratory, Cambridge, UK; ^64^ Department of Oncology, University of Cambridge, Strangeways Research Laboratory, Cambridge, UK; ^65^ Department of Pathology, University of Melbourne, Parkville, VIC, Australia; ^66^ Faculty of Medicine, University of Southampton, University Hospital Southampton, Southampton, UK; ^67^ Institute of Cancer Sciences, Unversity of Glasgow, Wolfson Wohl Cancer Research Centre, Beatson Institute for Cancer Research, Glasgow, UK; ^68^ Department of Health Research and Policy - Epidemiology, Stanford University School of Medicine, Stanford, CA, USA; ^69^ Department of Chronic Disease Epidemiology, Yale School of Public Health, New Haven, CT, USA; ^70^ Women's College Research Institute, University of Toronto, Toronto, ON, Canada; ^71^ Public Health Ontario, Toronto, ON, Canada; ^72^ Department of Epidemiology, Director of Genetic Epidemiology Research Institute, UCI School of Medicine, University of California Irvine, Irvine, CA, USA; ^73^ Department of Epidemiology, University of California Irvine, Irvine, CA, USA; ^74^ Department of Preventive Medicine, Keck School of Medicine, University of Southern California Norris Comprehensive Cancer Center, Los Angeles, CA, USA; ^75^ Women's Cancer, Institute for Women's Health, University College London, London, UK; ^76^ Department of Epidemology, University of Michigan School of Public Health, Ann Arbor, MI, USA; ^77^ Department of Pathology and Laboratory Diagnostics, the Maria Sklodowska-Curie Memorial Cancer Center and Institute of Oncology, Warsaw, Poland; ^78^ Department of Community and Family Medicine, Duke University Medical Center, Durham, NC, USA; ^79^ Cancer Control and Population Sciences, Duke Cancer Institute, Durham, NC, USA; ^80^ Department of Molecular Oncology, Moffitt Cancer Center, Tampa, FL, USA; ^81^ Ovarian Cancer Association Consortium

**Keywords:** polymorphisms, RNA editing, ovarian cancer risk

## Abstract

RNA editing in mammals is a form of post-transcriptional modification in which adenosine is converted to inosine by the adenosine deaminases acting on RNA (ADAR) family of enzymes. Based on evidence of altered ADAR expression in epithelial ovarian cancers (EOC), we hypothesized that single nucleotide polymorphisms (SNPs) in ADAR genes modify EOC susceptibility, potentially by altering ovarian tissue gene expression. Using directly genotyped and imputed data from 10,891 invasive EOC cases and 21,693 controls, we evaluated the associations of 5,303 SNPs in *ADAD1, ADAR, ADAR2, ADAR3*, and *SND1*. Unconditional logistic regression was used to estimate odds ratios (OR) and 95% confidence intervals (CI), with adjustment for European ancestry. We conducted gene-level analyses using the Admixture Maximum Likelihood (AML) test and the Sequence-Kernel Association test for common and rare variants (SKAT-CR). Association analysis revealed top risk-associated SNP rs77027562 (OR (95% CI)= 1.39 (1.17-1.64), *P*=1.0×10^−4^) in *ADAR3* and rs185455523 in *SND1* (OR (95% CI)= 0.68 (0.56-0.83), *P*=2.0×10^−4^). When restricting to serous histology (*n*=6,500), the magnitude of association strengthened for rs185455523 (OR=0.60, *P*=1.0×10^−4^). Gene-level analyses revealed that variation in ADAR was associated (*P*<0.05) with EOC susceptibility, with P_AML_=0.022 and P_SKAT-CR_=0.020. Expression quantitative trait locus analysis in EOC tissue revealed significant associations (*P*<0.05) with *ADAR* expression for several SNPs in ADAR, including rs1127313 (G/A), a SNP in the 3′ untranslated region. In summary, germline variation involving RNA editing genes may influence EOC susceptibility, warranting further investigation of inherited and acquired alterations affecting RNA editing.

## INTRODUCTION

Over the past decade it has been recognized that the complexity of higher organisms is related to the information stored in non-protein-coding regions of the genome. Such complexity may be attributed to a range of processing events and post-transcriptional modifications that affect the fate of RNA, including alternative splicing, 5′ capping, 3′ polyadenylation, and RNA editing [1-3]. The most common type of RNA editing in eukaryotes is site-selective hydrolytic deamination of adenosine into inosine (A-to-I) within double-stranded RNAs, and recent bioinformatic analyses and high-throughput sequencing efforts have revealed that A-to-I editing is widespread and alters non-coding and protein-coding sequences throughout the genome [4].

A-to-I editing is mediated by a family of *a*denosine *d*eaminases *a*cting on RNA (ADARs), and this process modulates expression of genes and biological pathways *via* several mechanisms [4]. Indeed, altered expression and/or activity of ADAR enzymes has been linked to a variety of conditions, including cardiovascular and neurological diseases and cancers [4]. Epithelial ovarian cancer (EOC) is the fifth leading cause of cancer death among women in the United States [5], and ADAR expression levels have been reported to be significantly higher in serum and peritoneal fluid from patients with EOCs compared with benign ovarian tumors [6, 7], suggesting ADARs may be useful biomarkers for the diagnosis and management of EOC.

We hypothesized that germline single nucleotide polymorphisms (SNPs) involving ADAR-related/RNA editing genes may contribute to EOC risk. The main purpose of this investigation was to determine whether SNPs in five ADAR genes (*ADAD1, ADAR, ADAR2, ADAR3*, and *SND1*) were associated with EOC susceptibility. We used data available from a large-scale genotyping collaboration involving 10,891 EOC cases and 21,693 controls from the international Ovarian Cancer Association Consortium (OCAC) [8]. We also sought to evaluate the overall contribution of each gene on EOC susceptibility and to determine whether candidate SNPs associated with altered expression of corresponding genes in EOC tumor tissue.

## RESULTS

### Study population

The study sample included 10,891 invasive EOC patients and 21,693 controls of European ancestry (Supplementary Table 1). Selected subject characteristics are shown in Table 1. The mean age at diagnosis for cases was 58.1 years, the mean age at interview for controls was 56.1 years. Cases were more likely than controls to be nulliparous and to have never used oral contraceptives. Most cases had serous histology (59.7%), distant stage (63.0%), and high-grade disease (58.9%).

**Table 1:** Characteristics of study participants (*N* = 32,584)

Variable	Cases (*n*= 10,891)	Controls (*n*= 21,693)
Age at diagnosis/interview(y), mean (SD)	58.1 (11.4)	56.1 (24.9)
History of pregnancy		
Yes	6021 (80.4)	15190 (87.9)
No	1318 (17.6)	1868 (10.8)
Unknown	149 (2.0)	217 (1.3)
Oral contraceptive use		
Ever	4017 (57.4)	10572 (63.3)
Never	2864 (41.0)	5900 (35.3)
Unknown	112 (1.6)	243 (1.5)
Histology		
Serous	6500 (59.7)	NA
Mucinous	696 (6.4)	
Endometrioid	1439 (13.2)	
Clear Cell	660 (6.1)	
Mixed Cell	369 (3.4)	
Other or unknown epithelial type	1227(11.3)	
Stage		
Localized	1425 (15.7)	NA
Regional	1838 (20.2)	
Distant	5721 (63.0)	
Unknown	103 (1.1)	
Grade		
I/II	2882 (32.8)	NA
III/IV	5174 (58.9)	
Other/Unknown	729 (8.3)	

### Variant-level association analysis and overlap with regulatory domains

**Figure 1:**
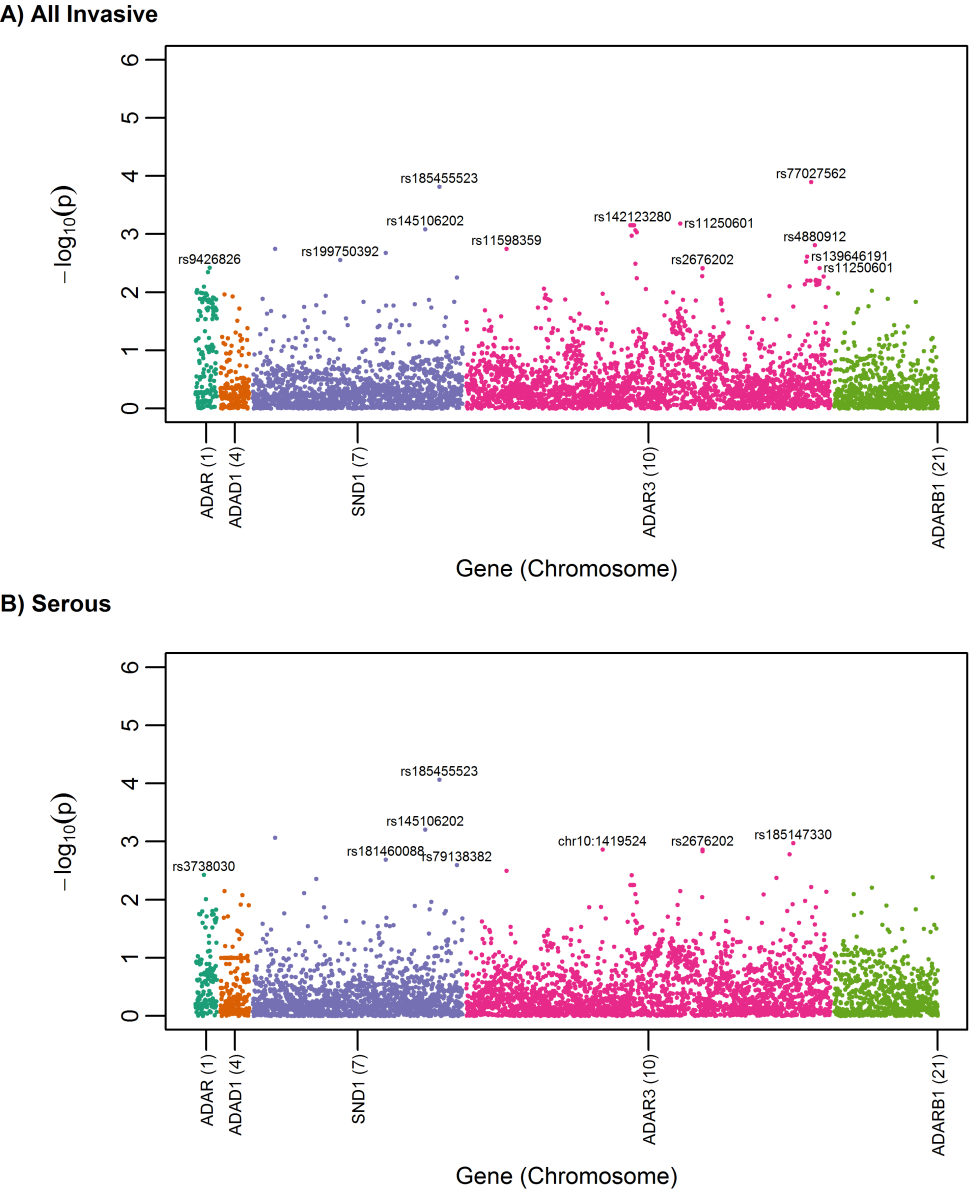
Manhattan plot for candidate RNA editing SNPs among a) all invasive cases (*n* = 10,891) *versus* controls (*n* = 21,693) and b) serous cases (*n* = 6,500) *versus* controls

**Figure 2:**
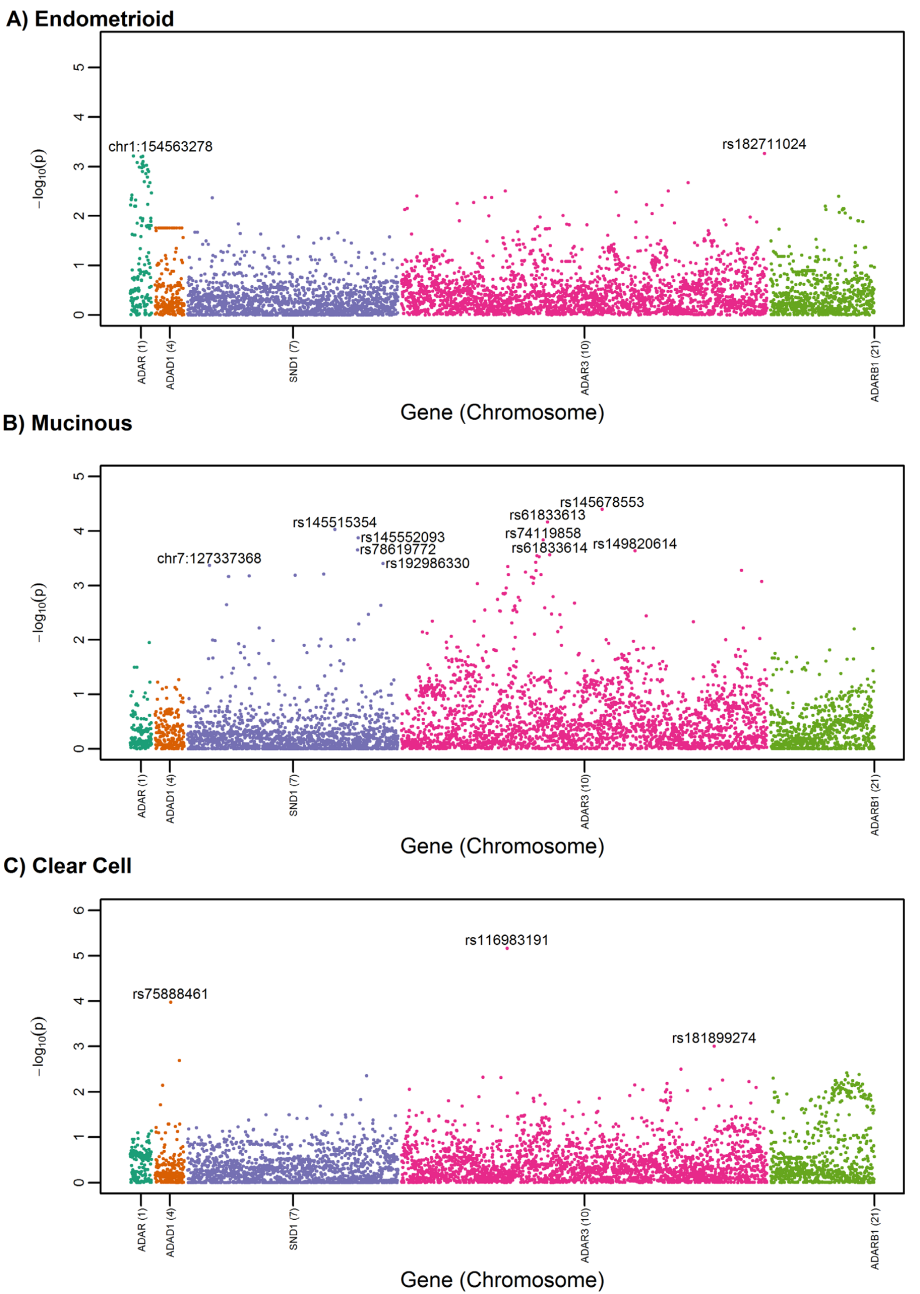
Manhattan plot for candidate RNA editing SNPs among a) endometrioid cases (*n* = 1,439) *versus* controls (*n* = 21,693), b) mucinous cases (*n* = 696) *versus* controls, and c) clear cell cases (*n* = 660) *versus* controls

SNP-level association analysis revealed top-ranked SNPs (defined as the top 5% of SNPs having the most statistically significant P values) in *ADAR, ADAR3*, and *SND1* in the all-histologies and serous-only analyses (Figure 1A and 1B). Table 2 summarizes association results for the most statistically significant SNPs overall or by serous histology (*P* < 4.0×10^−3^); associations were not significant after correction for multiple testing (FDR > 0.15). Most of the top-ranked variants were imputed, rare or low frequency (MAF < 0.05), and not part of a shared haplotype. rs77027562 (A>G; MAF = 0.009), the top risk-associated variant among all histologies (OR (95% CI) = 1.39 (1.17-1.64, *P* = 1.0 x10^−4^)), resides in an intron of *ADAR3. ADAR3* SNP rs77027562 and its proxies (r^2^>0.80) reside in genomic regions that overlap with regulatory domains, particularly enhancers in blood and brain (Table 3). The next top-ranked variant, *SND1* rs185455523 (T>A), was associated with a decreased EOC risk (OR (95% CI) = 0.68 (0.56-0.83), *P* = 1.5 x10^−4^), but this SNP and its proxies do not appear to overlap with regulatory domains. When analysis was restricted to the 6,500 patients with invasive serous adenocarcinomas, the magnitude of association was slightly attenuated for *ADAR3* rs77027562 (OR = 1.33, *P* = 6.1×10^−3^) and slightly stronger for *SND1* rs185455523 (OR = 0.60, P = 1×10^−4^). Exploratory analysis for the less common histologic subtypes (endometrioid (*n* = 1,439), mucinous (n = 696), and clear cell (*n* = 660)) revealed several SNP-level associations unique to each sub-type (Figure 2A-2C). For example, rs145678553-C in *ADAR3* is a rare variant (MAF = 0.0047) associated with an increased risk for mucinous EOC (OR (95%CI) = 3.46 (1.91-6.26), *P* = 3.99x 10^−5^), and rs116983191-A in *ADAR3* is a low-frequency variant (MAF = 0.044) associated with clear cell carcinoma (OR (95%CI) = 1.86 (1.42-2.43, *P* = 6.91 × 10^−6^). rs145678553-C was not represented in Haploreg. rs116983191-A is located in promoter and enhancer regions, but not in tissues relevant to ovarian cancer.

**Table 2:** Top-ranked RNA editing SNP-EOC risk associations among all histologies (*N* = 10,891) or serous histology (*N* = 6,500) versus controls (*N* = 21,693), sorted by gene and *p*-value

Gene	SNP	Alleles	MAF	Imputation accuracy R2	All histologies OR (95% CI)	*P*	FDR	Serous OR (95% CI)	*P*	FDR
ADAR	rs9426826	C>G	0.481	0.86	1.05 (1.02-1.09)	0.0038	0.76	1.04 (1-1.08)	0.0759	0.9996
	rs3738030	A>C	0.116	0.79	0.93 (0.89-0.98)	0.0080	0.83	0.91 (0.86-0.97)	0.0038	0.9996
ADAR3	rs77027562^a^	A>G	0.009	0.41	1.39 (1.17-1.64)	0.0001	0.34	1.33 (1.08-1.62)	0.0061	0.9996
	rs11250601	C>T	0.070	0.62	0.89 (0.83-0.95)	0.0007	0.34	0.9 (0.83-0.97)	0.0071	0.9996
	rs142123280^b^	A>G	0.001	0.48	2.08 (1.36-3.17)	0.0007	0.34	2.01 (1.23-3.29)	0.0056	0.9996
	rs4880912	T>C	0.200	0.82	1.07 (1.03-1.11)	0.0015	0.51	1.06 (1.01-1.11)	0.0267	0.9996
	rs11598359	C>T	0.005	0.45	0.68 (0.54-0.87)	0.0018	0.53	0.63 (0.47-0.86)	0.0032	0.9996
	rs6560760	C>T	0.025	0.59	1.17 (1.06-1.3)	0.0024	0.65	1.07 (0.95-1.22)	0.2688	0.9996
	rs2676202^c^	C>T	0.122	0.66	0.93 (0.88-0.98)	0.0038	0.76	0.9 (0.85-0.96)	0.0014	0.9996
	rs139646191	TAGAA>T	0.062	0.66	1.11 (1.03-1.18)	0.0038	0.76	1.07 (0.98-1.16)	0.1242	0.9996
	rs139812582	G>A	0.002	0.47	1.71 (1.15-2.54)	0.0078	0.83	2.03 (1.31-3.14)	0.0017	0.9996
	chr10:1419524	T>TGG	0.009	0.60	0.79 (0.65-0.95)	0.0106	0.83	0.68 (0.54-0.86)	0.0014	0.9996
	rs185147330	C>T	0.005	0.45	1.32 (1.05-1.67)	0.0176	0.86	1.54 (1.19-2)	0.0011	0.9996
SND1	rs185455523	T>A	0.008	0.56	0.68 (0.56-0.83)	0.0002	0.34	0.6 (0.46-0.77)	0.0001	0.45
	rs145106202^d^	G>C	0.009	0.87	0.73 (0.61-0.88)	0.0008	0.35	0.67 (0.53-0.84)	0.0006	0.9996
	rs181460088	C>T	0.008	0.80	0.73 (0.6-0.89)	0.0021	0.58	0.67 (0.52-0.86)	0.0020	0.9996
	rs199750392	G>GT	0.036	0.61	1.14 (1.05-1.25)	0.0028	0.71	1.11 (1-1.23)	0.0550	0.9996
	rs79138382	C>T	0.007	0.72	1.32 (1.09-1.61)	0.0056	0.83	1.42 (1.13-1.77)	0.0025	0.9996

Significant SNPs (P < 4.0×10^−3^) are listed and SNPs in LD (r^2^>0.60) with more significant SNP are not reported:

a) 7 SNPs in LD not reported

b) 12 SNPs in LD not reported

c) 1 SNP in LD not reported

d) 1 SNP in LD not reported

**Table 3:** HaploReg results for top-ranked ADAR3 SNP rs77027562 and its proxies from univariate analyses

Position (hg38)	LD (r2)	SNP (Ref>Alt)	MAF in EUR	Functional Annotation	CR	Promoter histone marks	Enhancer histone marks	DNAse site	Proteins bound	eQTL	Motifs Changed
Chr10:1688744	--	rs77027562 (A>G)	0.02	Intronic	No		BRN	BLD			ERalpha-a, RXRA, Zfp281
Chr10:1675149	0.94	rs12258319 (G>T)	0.98	Intronic	No		BLD				Pax-5
Chr10:1675875	0.94	rs7077743 (C>T)	0.98	Intronic	No		BLD				BDP1, CAC-binding-protein, HNF4, p300
Chr10:1676470	0.94	rs6560758 (T>C)	0.98	Intronic	No						
Chr10:1678882	0.94	rs10751814 (A>G)	0.98	Intronic	No						GR, Rad21
Chr10:1680294	0.94	rs7089727 (A>G)	0.98	Intronic	No		ESDR, IPSC, BLD, LNG	ESC, BLD	CTCF		
Chr10:1681695	0.94	rs6560759 (T>C)	0.98	Intronic	No		ESDR, BLD				Myc
Chr10:1687566	0.94	rs79784382 (T>A)	0.02	Intronic	No		BRN				Cphx, Duxl, HNF6, Hmx, Hoxa13, Pbx-1, Pbx3

Abbreviations: LD, linkage disequilibrium; MAF, minor allele frequency; EUR, European; CR, conserved region; eQTL, expression quantitative trait loci. Tissue groups: BRN, brain cells; BLD, blood and T-cells; ESDR, embryonic stem cell derived cells; IPSC, induced pluripotent stem cells; LNG, lung cell; ESC, embryonic stem cells. Proxies were defined as variants in LD (r^2^>0.8) with the index SNP rs77027562 (bolded) in 1000 genomes project Phase 1 data for Europeans. All data was accessed using HaploReg v4.1 available at: http://www.broadinstitute.org/mammals/haploreg/documentation_v4.1.html. Both conservation prediction algorithms, GERP and SiPhy-omega, were used. Only eQTLs for ADAR genes (5 genes) are given.

### Gene-level analyses

Gene-level analyses based on AML and SKAT-CR revealed that variation in *ADAR* was nominally associated (*P* < 0.05) with susceptibility to all invasive EOC, with *P* = 0.02 using both methods (Table 4). Histology-specific analyses revealed that *ADAR* variation was associated with endometrioid EOC susceptibility (P_SKAT-CR_ = 0.005/P_AML_ = 0.008). When using a Bonferroni threshold of 0.0025, only *ADAR3* variation was significantly associated with mucinous histology (P_SKAT-CR_ = 0.0016/P_AML_ = 0.031).

**Figure 3:**
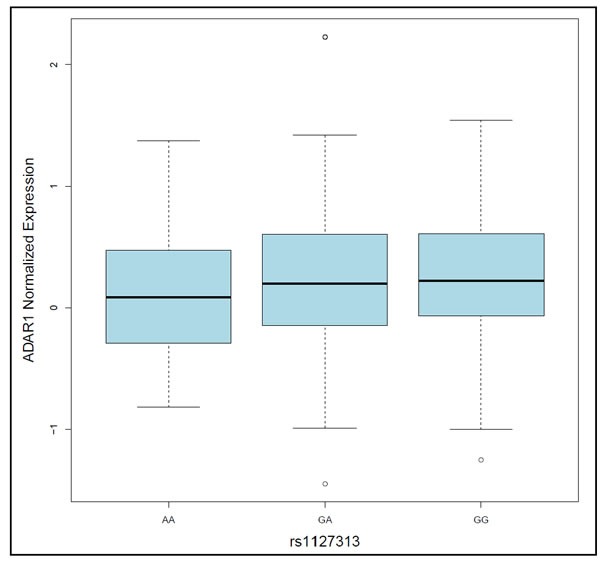
Box-plot showing that *ADAR1* tumor tissue expression differed, albeit only slightly, by rs1127313 genotype (*p* = 0.027)

To examine associations between genotype and gene expression for the 5 candidate RNA editing genes, expression quantitative trait locus (eQTL) analysis was performed using matched genotype and tissue expression data from The Cancer Genome Atlas (TCGA) high-grade serous adenocarcinoma tumors (https://tcga-data.nci.nih.gov/tcga/). eQTL analysis revealed statistically significant associations (*P* < 0.05) with *ADAR* expression for several SNPs in *ADAR*, including rs1127313 (G/A), a SNP in the 3′UTR within a putative miRNA binding site that was associated with susceptibility in all histologies (OR = 1.05, *P* = 0.009). rs1127313 is also in high LD (r^2^ = 0.86) with top *ADAR* risk SNP rs9426826 (see Table 2). *ADAR* tumor tissue expression was slightly higher among G allele carriers of rs1127313 compared to A allele carriers (*P* = 0.027; Figure 3). rs1127313 is also an eQTL for *ADAR* in whole blood (Supplementary Table 2), and lies in a genomic region with enhancer features and DNase I hypersensitivity site in several tissues, including ovary. Statistically-significant cis-eQTLs were not detected for SNPs in other candidate RNA editing genes.

**Table 4:** Association between RNA editing genes and EOC susceptibility.

Gene	Total N Markers (N Tested)	N Rare Markers (MAF<0.01)	N Common Markers (MAF≥0.01)	All Invasive		Serous		Endometrioid		Mucinous		Clear cell	
				P. SKAT-CR	P.AML Trend	P. SKAT-CR	P.AML Trend	P. SKAT-CR	P.AML Trend	P. SKAT-CR	P.AML Trend	P. SKAT-CR	P.AML Trend
**ADAD1**	210 (210)	98	112	0.698	0.749	0.300	0.433	0.054	0.179	0.857	0.804	0.696	0.635
**ADAR1**	155 (155)	50	105	0.020	0.022	0.110	0.101	0.005	0.008	0.943	0.841	0.780	0.411
**ADAR2**	754 (754)	301	4563	0.861	0.894	0.623	0.720	0.134	0.496	0.338	0.208	0.105	0.041
**ADAR3**	2656 (2654)	787	1867	0.216	0.266	0.334	0.541	0.587	0.470	0.002	0.031	0.234	0.502
**SND1**	1528 (1527)	764	763	0.630	0.809	0.703	0.376	0.919	0.895	0.632	0.204	0.773	0.535

## DISCUSSION

An emerging body of data suggest that defects in RNA editing may contribute to a range of human diseases, including cancer [2-4, 9-11]. The current large-scale collaboration represents the first comprehensive association study of germline variants involving RNA editing genes and susceptibility to epithelial ovarian cancer. At the SNP- level, the strongest associations were observed for SNPs in RNA editing genes *ADAR3* and *SND1*, but no associations reached genome-wide levels of statistical significance. Gene-level analyses highlighted *ADAR* and *ADAR3* as potential contributors to EOC susceptibility within the set of ADAR-related genes. Finally, positive eQTLs were also observed between ADAR genotype and ADAR expression in EOC tumor tissue.

Focused evaluations of RNA editing SNP-disease associations are limited [12], especially with cancer as an outcome, so it is not possible to compare our SNP findings to those of other studies of cancer risk. We are, however, unaware of GWAS hits in or near these genes. Several recent studies [2, 3] have evaluated the genomic landscape and clinical relevance of RNA editing in numerous human tissue types. These analyses used RNA-sequencing data from both tumor and normal samples profiled as part of TCGA Project. Striking differences in RNA-editing patterns were observed in tumors relative to matched normal tissues for 12 cancer types [2]. Further analyses revealed that altered RNA editing patterns in tumors correlated with *ADAR* expression, and that non-random, clinically-relevant RNA editing events (frequently located in noncoding RNAs, nonsynonymous sites, intronic regions, and non-*Alu* elements) correlated with tumor classification and patient survival and with increased cell survival and altered drug sensitivity [2, 3]. Interestingly, gene amplification-associated overexpression of *ADAR* was recently shown to enhance lung tumorigenesis and contribute to poor outcomes by affecting downstream RNA editing patterns [10]. As mentioned previously, ADAR expression levels have been reported to be significantly higher in serum and/or peritoneal fluid from patients with EOCs compared with benign ovarian tumors [6, 7]. Although high-grade serous EOCs from TCGA were not profiled as part of the aforementioned genomic investigations [18, 19], Haploreg 4.1 effectively integrates GTEX eQTL results for normal ovary.

Taken together with several lines of investigation from ovarian [6, 7] and other cancers [2, 3, 10] the current study suggests that ADARs (and *ADAR* in particular) may be useful biomarkers for the diagnosis and management of EOC. Thus, with replication, *ADAR* genotype status and/or expression level may serve as a risk factor for EOC. Indeed, we find that our top risk SNP in *ADAR*, rs9426826, has several proxy variants (r^2^>0.8, Supplementary Table 2) that are strongly associated with expression of this gene in blood (rs1127313: 7.23×10^−14^) and to a lesser extent, expression in high-grade serous EOC tumors (rs1127313: *P* = 0.027). Based on growing data which demonstrate the inhibition of tumor growth in the presence of ADAR inhibitors [13] and other therapeutic agents such as the IGFR-1R inhibitor BMS536924 and the MEK inhibitors CI1040 and trametinib [2], *ADAR* genotype and/or expression may help identify women whose tumors may respond to new combinations of therapies.

Strengths of the current study include the large sample size that primarily enabled detection of small effects for common variants, the relatively homogeneous population of EOC cases, and the multi-tiered genomic evaluation. However, this study was underpowered to detect the rare variants that were identified and is burdened by the low imputation quality. Additionally, the study is limited in that eQTL analysis did not permit adjustment for somatic copy number changes and DNA methylation status, factors that can influence transcript abundance and confound associations between germline polymorphisms and gene expression [14-16]. Moreover, it is possible that the top-ranked SNPs could potentially affect genes other than the RNA editing genes that drive candidate selection. Efforts to replicate these findings are needed; data will be available soon from a large, independent cohort of EOC cases genotyped by OCAC for this purpose (Amos et al, The OncoArray Consortium: a Network for Understanding the Genetic Architecture of Common Cancers (provisionally accepted, *CEBP*). Mechanistic studies to reveal how *ADAR* polymorphisms may affect oncogenic phenotypes will also be required, as will systematic investigations of the genomic landscape and clinical relevance of RNA editing in EOC using data from TCGA or other sources.

In summary, this study provides data to support the hypothesis that germline polymorphisms in ADAR related genes may influence gene expression and susceptibility to EOC. Further investigations are needed to determine whether inherited and acquired alterations affecting RNA editing serve as biological mechanisms to promote the development of EOC.

## MATERIALS AND METHODS

### Study population

A total of 41 studies (32 case-control and 9 case-only) from OCAC contributed to this investigation (Supplementary Table 1). Briefly, cases were women diagnosed with histologically confirmed primary invasive EOC (95%), fallopian tube cancer (1%), or primary peritoneal cancer (4%). Controls were women without cancer and with at least one intact ovary on the reference date. Individual studies were grouped into 26 case-control strata. All studies provided data on disease status, age at diagnosis/interview, self-reported racial group, and histologic subtype.

### Genotyping, quality control (QC), and imputation

Peripheral blood was the primary source of germline DNA and was collected in the course of clinical care or research at each of the participating sites. The candidate SNPs selected for the current investigation were genotyped using a custom Illumina Infinium iSelect Array as part of the international Collaborative Oncological Gene-environment Study (iCOGS), an effort to evaluate 211,155 genetic variants for association with cancer risk [17].

Briefly, OCAC genotyping was conducted at McGill University and Génome Québec Innovation Centre (Montréal, Canada) and Mayo Clinic Medical Genomics Facility. Each 96-well plate well contained 250ng genomic DNA (or 500 ng whole genome-amplified DNA). Raw intensity data files were sent to the COGS data coordination center at the University of Cambridge for genotype calling and QC using the GenCall algorithm. Sample and SNP quality control procedures have been described previously; in brief, samples were excluded with call rates < 95%, >1% discordance, < 80% European ancestry, or ambiguous gender, and SNPs were excluded with call rates < 95% or monomorphism [18, 19].

To improve genomic coverage and power [14], we imputed genotypes based on data from the 1000 Genomes Project (1KGP); we used IMPUTE2 version 2 after pre-phasing with SHAPEIT [20]. All 14 populations in the 1KGP were used as the reference. Before imputation, we excluded poorly performing SNPs according to the genotyping success rates, deviation from Hardy-Weinberg equilibrium (HWE) (*P* < 1×10^−7^), and replicate errors. To ensure the quality of the imputed genotypes, maximum likelihood genotype imputation was carried out and an estimate of the squared correlation between the imputed and true genotypes was calculated. Imputation quality is significantly decreased for low and rare frequency variants [21]. To be more inclusive of rare variants, we considered imputed SNPs with an r^2^> 0.40 as well-imputed [22] and included them in our analyses. The average imputation quality for included variants is detailed in Supplementary Table 4, overall and by MAF categories.

### Gene and SNP selection

Five candidate genes were chosen for this study based on published literature which directly showed or suggested roles in the regulation of A-to-I RNA editing [1, 4, 23]. The genes included adenosine deaminase domain containing 1 (*ADAD1*), adenosine deaminase, RNA-specific (*ADAR/ADAR1*), adenosine deaminase, RNA-specific, B1 (*ADARB1/ADAR2*), adenosine deaminase, RNA-specific, B2 (*ADAR3/ADARB2*), and staphylococcal nuclease and Tudor domain containing 1 (*SND1*). In total, 5,303 SNPs in the 5 genes, 77 genotyped directly and 5,226 imputed, were available for statistical analysis.

### Population stratification

HapMap DNA samples from European (CEU, *n* = 60), African (YRI, *n* = 53) and Asian (JPT+CHB, *n* = 88) populations were also genotyped as part of the same custom Illumina iSelect Array. The program LAMP [24] was used to estimate intercontinental ancestry based on the HapMap (release no. 23) genotype frequency data for these three populations. Eligible subjects with greater than 90 percent European ancestry were defined as European (*n* = 39,773). We then used a set of 37,000 unlinked autosomal markers to perform principal components analysis within each major population subgroup. To enable this analysis on very large sample sizes we used an in-house program written in C++ using the Intel MKL libraries for eigenvectors (available at http://ccge.medschl.cam.ac.uk/software/).

### Statistical analysis

Descriptive statistics were calculated in terms of means and standard deviations for continuous variables and frequencies and percents for categorical variables. The primary association analysis focused on individuals of European ancestry. Unconditional logistic regression was used to estimate odds ratios (OR) and their 95% confidence intervals (CI) between genotype and case status under a log-additive genetic model, with adjustment for the first five principal components representing sub-European ancestry. Due to the heterogeneous nature of EOC, subgroup analyses were conducted to estimate genotype-specific odds ratios by histologic subtype: serous, endometrioid, mucinous, and clear cell carcinomas. False discovery rates (FDR) [25] were used to adjust for multiple comparisons, and FDR of 15% was used to declare significance.

Two methods of gene-level evaluations were also conducted to combine association evidence from SNPs within each gene evaluated: the Admixture Maximum Likelihood (AML) Test [26] and the Sequence-Kernel Association test for the combined effect of common and rare variants (SKAT-CR) [27]. AML is an approach that simultaneously examines the global null hypothesis (of no SNP-outcome associations) and estimates the proportion of underlying false hypotheses. The AML uses univariate SNP-level results to calculate the AML Cochran-Armitage Trend test. Compared to other methods, AML has been shown to have similar or higher statistical power to detect associations except under the unlikely scenario that greater than 20% of all variants are associated with the outcome [26]. SKAT-CR evaluates the cumulative effect of rare and common variants, but does not consider low-frequency variants. These gene-level approaches were undertaken to complement SNP-level findings, and aimed to reduce the degrees of freedom, avoid model-fitting issues due to multicollinearity from LD, and to improve statistical power. The Bonferroni method was used to account for multiple comparisons.

Expression quantitative trait locus (eQTL) analysis was performed to examine for association between genotype (*n* = 5,303, imputed as above in *n* = 5 genes) and corresponding gene expression for the 5 candidate RNA editing genes. Matched genotype and gene expression profiling data were obtained for 402 high-grade serous EOC samples evaluated in the Cancer Genome Atlas (TCGA) Project using previously described methods [19]. Briefly, germline genotypes and matched tumor gene expression data were downloaded from the TCGA data portal. To conduct the eQTL analysis, we used germline genotypes of SNPs/proxies as independent variables and expression levels as traits. Expression levels between minor allele carriers versus non-carriers were compared using the Wilcoxon rank sum statistic. Haploreg v4.1http://www.broadinstitute.org/mammals/haploreg/haploreg.php) [28] was used to evaluate the putative function of candidate SNPs.

## SUPPLEMENTARY MATERIALS TABLES






