# Supplementary material for: Inherited variants affecting RNA editing may contribute to ovarian cancer susceptibility: results from a large-scale collaboration
Source: Oncotarget. 2016 Jul 12;7(45):72381–94. doi: 10.18632/oncotarget.10546 (PMC5340123; doi:10.18632/oncotarget.10546)
Supplement: Supplementary file 1 [file oncotarget-07-72381-s001.pdf]

Inherited variants affecting RNA editing may contribute to ovarian cancer susceptibility: results from a large-scale collaboration

Supplementary Material

Supplementary Table 1: Description of individual OCAC studies and case-control sets included in the analysis of RNA editing SNPs and EOC susceptibility[1].

For Table S1, please see the attached file

Supplementary Table 2: HaploReg view of top *ADAR* eQTL SNP rs1127313 and proxies.

For Table S2, please see the attached Excel file
