# Supplementary material for: Inherited variants affecting RNA editing may contribute to ovarian cancer susceptibility: results from a large-scale collaboration
Source: Oncotarget. 2016 Jul 12;7(45):72381–94. doi: 10.18632/oncotarget.10546 (PMC5340123; doi:10.18632/oncotarget.10546)
Supplement: Supplementary file 2 [file oncotarget-07-72381-s002.docx]

Supplementary Table 1: Description of individual OCAC studies and case-control sets included in the analysis of RNA editing SNPs and EOC susceptibility^1^.

| **Study Name(s)** | **Study abbreviation(s)** | **Study Location** | **Study Type** | **Total Number of Subjects^2^** | | |
| --- | --- | --- | --- | --- | --- | --- |
|  |  |  |  | **All Cases** | **Serous cases** | **Controls** |
| Australian Ovarian Cancer Study/Australian Cancer Study (Ovarian Cancer)/Melbourne Collaborative Cohort Study | AOCS/AUS/MCC | Australia | Population based case-control/nested case-control | 942 | 586 | 1043 |
| Bavarian Ovarian Cancer Cases and Controls | BAV | Southeast Germany | Population based case-control | 93 | 56 | 143 |
| Belgian Ovarian Cancer Study | BEL | Belgium, University Hospital Leuven | Hospital based Case-control | 275 | 195 | 1349 |
| Diseases of the Ovary and their Evaluation/Oregon Ovarian Cancer Registry | DOV/ORE | USA: 13 counties in western Washington state/Portland, ORE | Population based case-control/Case only | 959 | 568 | 1487 |
| German Ovarian Cancer Study/ Dr. Horst Schmidt Kliniken | GER/HSK | Germany: Baden-Württemberg and Rhineland-Palatinate/ Germany | Population based case-control/Case only | 333 | 202 | 413 |
| Hawaii Ovarian Cancer Case-Control Study | HAW | USA: Hawaii | Population based case-control | 60 | 38 | 157 |
| Hannover-Jena Ovarian Cancer Study | HJO | Germany | Hospital based Case-control | 261 | 140 | 273 |
| Hannover-Minsk Ovarian Cancer Study | HMO | Belarus | Hospital-based Case-control | 142 | 50 | 138 |
| Helsinki Ovarian Cancer Study | HOC | Helsinki, Finland | Case-control | 217 | 113 | 447 |
| Hormones and Ovarian Cancer PrEdiction (HOPE) Study/Gilda Radner Familial Ovarian Cancer Registry | HOP/GRR | USA: West Pennsylvania, Northeast Ohio, West New York/USA | Population based Case-control/Case-only | 765 | 450 | 1464 |
| Women’s Cancer Program at the Samuel Oschin Comprehensive Cancer Institute/ Los Angeles County Case-control studies of Ovarian Cancer-1 | LAX/USC | USA: Southern California/Los Angeles County | Case only/Population based Case-control | 966 | 663 | 1047 |
| MALignant OVArian Cancer/ Danish Pelvic Mass Study | MAL/PVD | Denmark | Population based Case-control/ Case only | 609 | 400 | 829 |
| MD Anderson Ovarian Cancer Study | MDA | USA: Texas | Hospital based Case-control | 313 | 190 | 384 |
| Memorial Sloan-Kettering Cancer Center | MSK | USA: New York City | Hospital-based Case-control | 467 | 382 | 593 |
| North Carolina Ovarian Cancer Study | NCO | USA: Central and eastern North Carolina (48 counties) | Population based Case-control | 269 | 147 | 172 |
| New England Case Control Study | NEC | USA: New Hampshire and Eastern Massachusetts | Population based Case-control | 634 | 371 | 980 |
| Nurses’ Health Study | NHS | USA | Cohort/ Nested case-control | 138 | 77 | 455 |
| New Jersey Ovarian Cancer Study/ Mayo Clinic Ovarian Cancer Study/ Toronto Ovarian Cancer Study | NJO/MAY/TOR | USA/Canada: New Jersey Minnesota and 6-state surrounding region/Canada: Providence of Ontario | Population-based Case-control/ Population-based case-control/ Population based | 197 | 117 | 265 |
| University of Bergen, Haukeland University Hospital, Norway | NOR | Norway | Case-control | 234 | 135 | 371 |
| Nijmegen Ovarian Cancer Study | NTH | Eastern part of the Netherlands | Case-control | 255 | 116 | 323 |
| Ovarian Cancer in Alberta and British Columbia | OVA | Alberta and British Columbia, Canada | Population-based Case-control | 621 | 344 | 748 |
| Poland Ovarian Cancer Study/Warsaw Ovarian Cancer Study | POC/POL/WOC | Poland: Szcesin, Poznan, Opole, and Rzeszow/ Warsaw and central Poland | Hospital-based Case-control/ Hospital-based Case-control | 666 | 352 | 807 |
| Study of Epidemiology and Risk Factors in Cancer Heredity | SEA | UK: East Anglia and West Midlands | Population based Case-control | 284 | 173 | 6023 |
| Family Registry for Ovarian Cancer and Genetic Epidemiology of Ovarian Cancer | STA | USA: Six counties in the San Francisco Bay area | Population based Case-control | 251 | 154 | 313 |
| University of California Irvine Ovarian Study | UCI | USA: Southern California (Orange and San-Diego, Imperial Counties) | Population based Case-control | 277 | 166 | 367 |
| United Kingdom Ovarian Cancer Population Study/ Royal Marsden Hospital Ovarian Cancer Study/ UK Familial Ovarian Cancer Registry/ Southampton Ovarian Cancer Study/Scottish Randomized Trial in Ovarian Cancer | UKO/RMH/  UKR/SOC/SRO | United Kingdom (England, Wales and Northern Ireland)/UK:London/  UK:National/UK, Wessex region/ Coordinated through clinical trials unit, Glasgow UK patients recruited world-wide | Population based Case-control/ Hospital based Case only/Case only Familial Register/ Case only Hospital based/ Case only clinical trial | 663 | 315 | 1103 |
| **TOTAL** |  |  |  | **10,891** | **6,500** | **21,693** |

1 Studies combined into single case controls sets (AOCS+ACS+MCC; DOV+ORE; HOP+GRR; GER+HSK; NJO+MAY+TOR; LAX+USC; MAL+PVD; POC +POL+WOC; UKO+RMH+UKR+SOC+SRO)

2 Totals represent the number of subjects passing genotyping quality control criteria.
